# Community resilience and associated factors in Fort McMurray a year after the devastating flood

**DOI:** 10.1192/j.eurpsy.2023.2102

**Published:** 2023-07-19

**Authors:** G. Obuobi-Donkor, E. Eboreime, R. Shalaby, B. Agyapong, M. Adu, E. Owusu, W. Mao, V. I. O. Agyapong

**Affiliations:** 1 Psychiatry, Dalhousie University, Halifax; 2 Psychiatry, University of Alberta, Edmonton, Canada

## Abstract

**Introduction:**

A natural disaster like flooding causes loss of properties and evacuation and effective mental health. Resilience after natural disasters is a crucial area of research which needs attention.

**Objectives:**

To explore the prevalence and associated factors of low resilience a year after the 2020 floods in Fort McMurray.

**Methods:**

A cross-sectional study was conducted in Fort McMurray using online surveys. The data were analyzed with SPSS version 25 using univariate analysis with the chi-squared test and binary logistic regression analysis.

**Results:**

The prevalence of low resilience was 37.4%. Respondents under 25 years were nearly 26 times more likely to show low resilience (OR= 0.038; 95% CI 0.004 - 0.384). Responders with a history of depression and anxiety (OR= 0.212; CI 95% 0.068-0.661) were nearly four to five times more likely to show low resilience. Similarly, respondents willing to receive mental health counselling (OR=0.134 95%CI: 0.047-0.378) were 7.5 times more likely to show low resilience. Participants residing in the same house before the flood were almost 11 times more likely to show low resilience (OR=0.095; 95% CI 0.021- 0.427), and support from the Government of Alberta was a protective factor.

**Conclusions:**

The study showed demographic, clinical, and flood-related variables contributing to low resilience. Receiving support from the Government was shown to be a protective factor against low resilience. More robust measures must be in place to promote normal to high resilience among flood victims in affected communities.

**Disclosure of Interest:**

None Declared

